# Interaction Prediction Optimization in Multidisciplinary Design Optimization Problems

**DOI:** 10.1155/2014/698453

**Published:** 2014-02-19

**Authors:** Debiao Meng, Xiaoling Zhang, Hong-Zhong Huang, Zhonglai Wang, Huanwei Xu

**Affiliations:** School of Mechanical, Electronic, and Industrial Engineering, University of Electronic Science and Technology of China, No. 2006, Xiyuan Avenue, West Hi-Tech Zone, Chengdu, Sichuan 611731, China

## Abstract

The distributed strategy of Collaborative Optimization (CO) is suitable for large-scale engineering systems. However, it is hard for CO to converge when there is a high level coupled dimension. Furthermore, the discipline objectives cannot be considered in each discipline optimization problem. In this paper, one large-scale systems control strategy, the interaction prediction method (IPM), is introduced to enhance CO. IPM is utilized for controlling subsystems and coordinating the produce process in large-scale systems originally. We combine the strategy of IPM with CO and propose the Interaction Prediction Optimization (IPO) method to solve MDO problems. As a hierarchical strategy, there are a system level and a subsystem level in IPO. The interaction design variables (including shared design variables and linking design variables) are operated at the system level and assigned to the subsystem level as design parameters. Each discipline objective is considered and optimized at the subsystem level simultaneously. The values of design variables are transported between system level and subsystem level. The compatibility constraints are replaced with the enhanced compatibility constraints to reduce the dimension of design variables in compatibility constraints. Two examples are presented to show the potential application of IPO for MDO.

## 1. Introduction

The Multidisciplinary Design Optimization (MDO) has received considerable attentions because of the increasing system complexity and the discipline interactions. In a multidisciplinary system, strong interactions between disciplines require taking advantage of the parallel design process [1]. A coordination strategy is applied to drive the design variables in different disciplines towards the optimum for the original problem [2]. Generally, there are two kinds of MDO coordination methods, nonhierarchical manner, and hierarchical manner [3–5]. For the nonhierarchical manner, using Multidisciplinary Feasible (MDF) approach, the Multidisciplinary Analysis (MDA) is performed multiple times via the fixed-point iteration [6]. The MDA is necessary at both each iteration and every point where the derivatives are to be evaluated, if a gradient-based method is used. Thus MDF is expensive in realistic application [7]. Using the All-at-Once (AAO) approach, the feasibility can be guaranteed when the optimization converges. The optimizer provides analysis models with inputs and outputs, and analysis models establish the discrepancies for the estimated inputs and outputs. The computational burden to maintain the feasible is relieved; however, the number of constrains for the discrepancy of the interdisciplinary variables is increased [8]. The Individual Discipline Feasible (IDF) method maintains the discipline feasibility when the optimizer drives disciplines to the multidisciplinary feasibility and optimality. Concurrent and independent discipline analyses are available in IDF. However, there will be a large number of optimization variables when applying IDF [9]. The specific analysis variables that represent communication between disciplines are treated as optimization variables [10]. Using the Concurrent Subspace Optimization (CSSO) method, the design variables are assigned to individual disciplines by the Global Sensitivity Equation (GSE). Each discipline performs a separate optimization by operating on its own discipline design variables. The coordination problem is solved by GSE and the optimum sensitivity derivatives with respect to parameters [11]. However, no two disciplines are allowed to operate on the same design variables in CSSO [12]. For the hierarchical manner, the Collaborative Optimization (CO) method decomposes the optimization problem and eliminates the need for disciplines by associating the design variables and linking variables with the system-level design variables. The system-level design variables may be shared between disciplines. These shared variables converge to common values by a coordination problem ultimately [13–15]. However, if there are many linking variables between disciplines, it is difficult to apply CO. It is because the extreme number of linking variables may make the consistence constraints failed [16]. The Bi-Level Integrated System Synthesis (BLISS) method uses a gradient-guided method to improve the system design, alternating between the discipline design variables and the system level design variables. The system level optimization problem deals with a small number of linking variables, while the discipline optimization problems deal with a relative larger number of discipline design variables [17]. However, BLISS has the disadvantage that it needs expensive sensitivity information for GSE and the optimum sensitivity analysis [18, 19].

Although many MDO methods have been proposed, new strategies are also needed to allow designers to select an appropriate method among various MDO methods. In this paper, the Interaction Prediction Optimization (IPO) is introduced based on the Interaction Prediction method (IPM) strategy. There are the system level and the subsystem level in IPO. At the system level, the discipline design variables are fixed and treated as the design parameters, while the interacting variables (including the shared variables and linking variables) are operated as design variables. At the subsystem level, the discipline design variables are operated as design variables, while the interacting variables are treated as the design parameters. The discipline objectives are optimized at the subsystem level in parallel. The system controller updates the interaction design variables for the subsystem optimization problems.

This paper is organized as follows. In Section 2, the theory of control and coordination in large-scale systems is introduced and the IPM strategy is briefly reviewed. In Section 3, the MDO problem is given. In Section 4, the IPO strategy is discussed in detail, including the formulation and the procedure. In Section 5, two examples are used to illustrate the effectiveness of the proposed method, followed by conclusions in Section 6.

## 2. Large-Scale Systems and the Interaction Prediction Method

**Figure 1:**
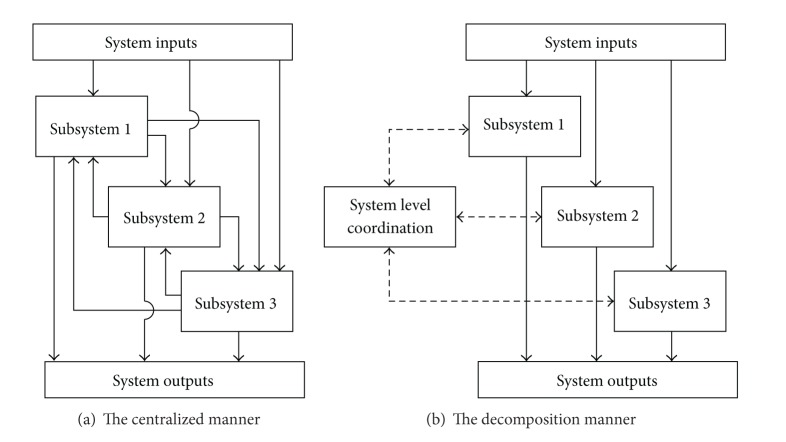
The large-scale system control problems.

For the large-scale systems, the control process is not conducted in a centralized manner because of the high complexity and the dimensionality problem. The decomposition methods can be used to solve the control problems as shown in Figure 1. In this way, a large-scale system is decomposed into several interconnected subsystems. The original control problem is redefined as the system level control problem and the subsystem level control problems. Each subsystem solves its own control problem and the system level coordination monitors and coordinates these subsystem problems.

**Figure 2:**
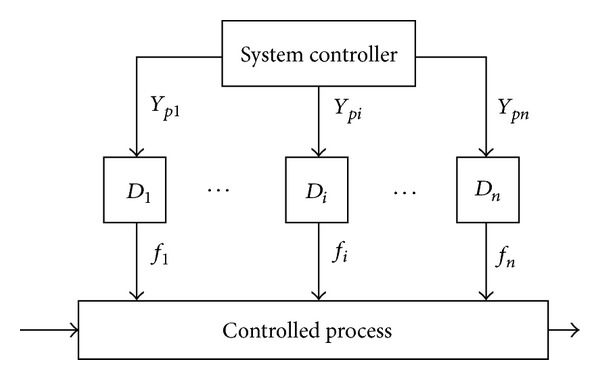
The framework of the IPM.

As one of the large-scale system control method, IPM is introduced here. Using IPM, the system is decomposed into *n* subsystems denoted by *D*_1_,…, *D*_*n*_. As shown in Figure 2, there are two levels such as the system level, which manages the overall process using the control variables *Y*_*pi*_, and the subsystem level, which manipulates the subsystem control problem and outputs the subsystem control solutions *f*_*n*_, *i* = 1 ~ *n*. The control process is conducted until subsystem control problems are solved and the compatibility conditions between the subsystems are satisfied. The details of IPM are given in [20].

## 3. MDO Problems

In MDO problems, each discipline possesses a certain degree of autonomy but also depends on other disciplines through the interacting variables. The outputs of one discipline may become inputs of other disciplines. The formulation of MDO problem is given in

(1) min⁡Xs,Xi,Yji,Yij f=f(Xs,Xi,Yji,Yij)s.t.      gi(Xs,Xi,Yji,Yij)>0        hi(Xs,Xi,Yji,Yij)=0        Yij=Yij(Xs,Xi,Yji)        Xmin⁡≤Xi≤Xmax⁡ Xsmin⁡≤Xs≤Xsmax⁡        Yijmin⁡≤Yij≤Yijmax⁡ Yjimin⁡≤Yji≤Yjimax⁡     i,j=1,2,…,n i≠j,

where *f*(·) denotes the system objective; *g*(·) denotes inequality constraints; *h*(·) denotes equality constraints; **X**_*s*_ denotes a vector of shared design variables; **X**_*i*_ denotes a vector of discipline design variables of the *i*th discipline; **Y**_*ji*_ denotes a vector of linking variables, which are inputs of the *i*th discipline and outputs of the other disciplines *j*; **Y**_*ij*_ denotes a vector of linking variables, which are inputs of the other disciplines *j* and outputs of the *i*th discipline; *n* denotes the total number of the disciplines; **Y**_*ij*_ = **Y**_*ij*_(**X**_*s*_, **X**_*i*_, **Y**_*ji*_) denotes the coupled information between the coupled disciplines.

## 4. The Interaction Prediction Optimization for MDO Problems

Combining the control and coordination strategy of IPM and the distributed design strategy of CO, the IPO method is proposed to solve the MDO problem in (1). Like the architecture of CO, there are the system level and the subsystem level in IPO. The system level minimizes the system objective with the design parameters which are the solutions of the discipline design variables **X**_*i*_ from the subsystem level. Then the system level determines the values of interaction variables **X**_*s*_, **Y**_*ji*_, and **Y**_*ij*_ for the disciplines at the system level. The optimization problem at the system level is given in

(2) Given    Xik−1Find       Xsk,Yjik,Yijkmin⁡Xsk,Yjik,Yijk f=f(Xsk(Xik−1),Yjik(Xik−1),Yijk(Xik−1))s.t.      gi(Xsk(Xik−1),Yjik(Xik−1),Yijk(Xik−1))>0       hi(Xsk(Xik−1),Yjik(Xik−1),Yijk(Xik−1))=0       Xsmin⁡≤Xs≤Xsmax⁡ Yi•min⁡≤Yi•≤Yi•max⁡       Y•imin⁡≤Y•i≤Y•imax⁡i,j=1,2,…,n i≠j,

where *k* presents the *k*th cycle in the optimization process.

The subsystem level tries to find the solutions of the discipline design variables **X**_*i*_. In the discipline optimization problems, interaction variables **X**_*s*_^*k*^, **Y**_*ji*_^*k*^, and **Y**_*ij*_^*k*^ are the design parameters. The discipline objectives are optimized in parallel and simultaneously. The discipline optimization problems at the subsystem level are given in

**Figure 3:**
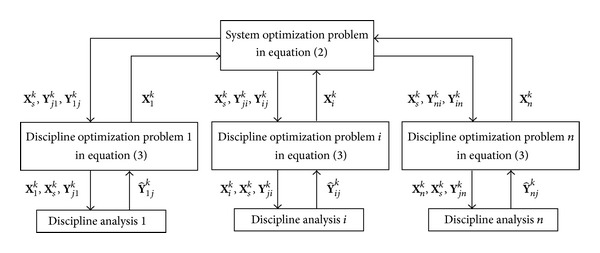
The framework of the IPO.

(3) Given     Xsk,Yjik,YijkFind        Xikmin⁡Xsk,Yjik,Yijk fi=fi(Xik(Xsk,Yjik,Yijk))s.t.      gi(Xik(Xsk,Yjik,Yijk))>0       hi(Xik(Xsk,Yjik,Yijk))=0       Ji=∑j=1,j≠in(Y^ijk−Yijk)2≤ε      Ximin⁡≤Xik≤Ximax⁡i,j=1,2,…,n i≠j,

where ${\hat{Y}}_{ij}^{k}$ is the outputs of the discipline analysis, ${\hat{Y}}_{ij}^{k}=Y_{ij}^{k}\left(X_{i}^{k},X_{s}^{k},Y_{ji}^{k}\right)$; *J*_*i*_ is the enhanced compatibility constraint; and *ε* is a very small positive number. The framework of IPO is given in Figure 3.

The procedure of IPO includes the following steps.

**Step 1**

Set initial values for design variables **X**_*i*_^0^, **X**_*s*_^0^, **Y**_*ji*_^0^, and **Y**_*ij*_^0^, *k* = 1.

**Step 2**

Solve the system optimization problem in (2) at the system level. The discipline design variables **X**_*i*_^0^ are used as design parameters. Obtain the *k*th cycle system solutions **X**_*s*_^*k*^, **Y**_*ji*_^*k*^, and **Y**_*ij*_^*k*^, and then send them to the subsystem level.

**Step 3**

Solve the discipline optimization problems in (3) at the subsystem level. The distributed design strategy of IPO allows discipline optimizations to be conducted in parallel and simultaneously. The interaction design variables **X**_*s*_^*k*^, **Y**_*ji*_^*k*^, and **Y**_*ij*_^*k*^ and the output of discipline analysis ${\hat{Y}}_{ij}^{k}$ are used as design parameters. The enhanced compatibility constraints *J*_*i*_ are used to diminish the discrepancy between **Y**_*ij*_^*k*^ and ${\hat{Y}}_{ij}^{k}$ during the discipline optimization process.

**Step 4**

Check the convergence. Calculate *G* = (**Y**_*ij*_^*k*^−**Y**_*ij*_^*k*−1^)^2^ + (**Y**_*ji*_^*k*^−**Y**_*ji*_^*k*−1^)^2^ + (**X**_*s*_^*k*^−**X**_*s*_^*k*−1^)^2^ + (**X**_*i*_^*k*^−**X**_*i*_^*k*−1^)^2^. If *G* ≤ *ε*, the values of the system objective and the discipline objectives are stable, and go to Step 5; otherwise, set *k* = *k* + 1 and go to Step 2.

**Step 5**

Stop the optimization process. Output the solutions **Y**_*i*•_^*k*^, **Y**_•*i*_^*k*^, **X**_*s*_^*k*^, and **X**_*i*_^*k*^.

**Figure 4:**
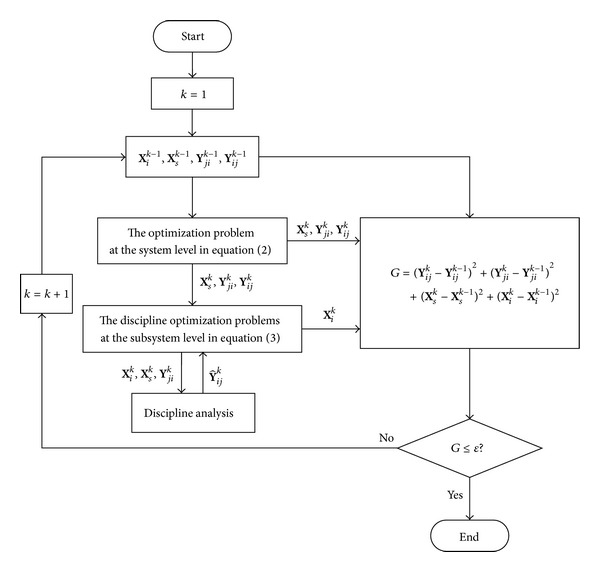
The flowchart of IPO.

The flowchart of IPO is given in Figure 4.

## 5. Examples

In this section, we use two examples to show the application of the proposed method. The efficiency and accuracy of the proposed method are compared with CO. The solutions from MDF are considered as the correct results.

### 5.1. Mathematical Example

The mathematical problem is provided as a simple test problem for testifying the proposed method. The design optimization problem is given as

(4) min⁡ f=(y12−1)2+x12+x22+(y21−2)2+x32  s.t.    −1≤x1≤1, −1≤x2≤1, −5≤x3≤5      −5≤y12≤5, −5≤y21≤5   y12=x1−x2+2y21, y21=x3−y12,

where *f* is the system objective and *x*_11_, *x*_12_, *x*_21_, *y*_12_, and *y*_21_ are design variables.

**Figure 5:**
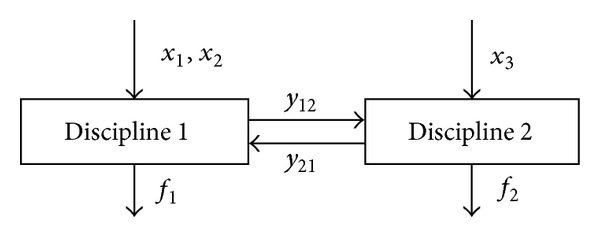
The MDO problem of the mathematical example.

Here, this problem is modified into a MDO problem including two disciplines shown in Figure 5. In the modified problem, *f*_1_ and *f*_2_ are the discipline objectives, two coupled variables *y*_12_ and *y*_21_ affect each other.

The optimization problems using IPO are provided in (5), (6), and (7).

(1) System optimization problem at the system level is as follows:
  (5) min⁡ f=(y12−1)2+x12+x22+(y21−2)2+x32  s.t.    −5≤y12≤5, −5≤y21≤5       design variables=[y12,y21]       design parameters=[x1,x2,x3].
(2) Discipline optimization problem 1 at the subsystem level is as follows:
  (6) min⁡ f1=(y12−1)2+x12+x22  s.t.    −1≤x1≤1, −1≤x2≤1       J1=(y^12−y12)2≤ε,       design variables=[x1,x2]       design parameters=[y12,y21].
(3) Discipline optimization problem 2 at the subsystem level is as follows:
  (7) min⁡ f2=(y21−2)2+x32  s.t.    −5≤x3≤5, y^21=x3−y12   J2=(y^21−y21)2≤ε       design variables=[x3]       design parameters=[y12,y21].

The optimization processes are conducted at two different initial points, (*x*_1_, *x*_2_, *x*_3_, *y*_12_, *y*_21_) = (0,0, 0,0, 0) and (*x*_1_, *x*_2_, *x*_3_, *y*_12_, *y*_21_) = (−1,1, −1,1, −1). The solutions are compared in Table 1. Here the compatibility constraints in IPO and CO are *J* < 0.001. *n*_*s*_ is the number of function calls at the system level; *n*_1_ and *n*_2_ are the numbers of function calls of discipline 1 and 2 at the subsystem, respectively. The solutions of two methods have the same accuracy. However, the numbers of function calls at both the system level and the subsystem level using IPO are less than that using CO. The reason is that the discipline objectives in IPO are different to the discipline objectives in CO and the enhanced compatibility constraints in IPO are simpler than the original compatibility constraints in CO.

### 5.2. Speed Reducer Design

**Figure 6:**
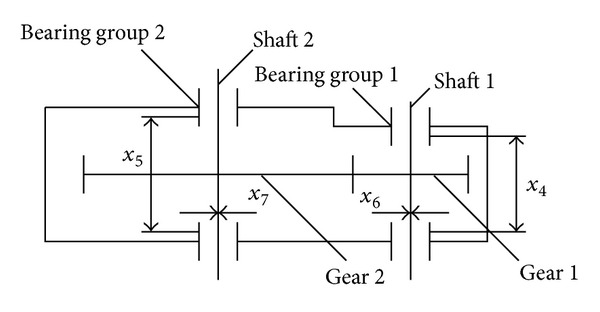
Design of the speed reducer.

This problem is an artificial NASA MDO test example in [7]. There are the power input discipline and the power output discipline shown in Figure 6.

The optimization problem is defined as

(8) min⁡ f(speed reducer overall volume)  s.t.    g1(bending stress of gear tooth)≤0       g2(contact stress of gear tooth)≤0       g3,g4(transverse deflection of shafts 1,2)≤0       g5,g6(stresses in shafts 1,2)≤0       g7~g9(dimensional restrictions)≤0       g10,g11(demensional requirements for shafts 1,2)≤0.

The optimization problems using IPO are provided in (9), (10) and (11).

(1) System optimization problem at the system level is as follows:
  (9) min⁡ f=0.7854x1x22(3.333x32+14.933x3−43.0934)          −1.508x1(x62+x72)+7.477(x63+x73)          +0.7854(x4x62+x5x72)s.t.     g1=27(x1x22x3)−1≤0 g2=397.5(x1x22x32)−1≤0      g3=1.93x43(x2x3x64)−1≤0 g4=1.93x53(x2x3x74)−1≤0      g5=A1B1−1100≤0 g6=A2B2−850≤0      g7=x2x3−40≤0 g8=x1x2−12≤0      g9=−x1x2+5≤0     2.6≤x1≤3.6 0.3≤x2≤1.0 17≤x3≤28      design variables=[x1,x2,x3]      design parameters=[x4,x5,x6,x7].
(2) Power input discipline optimization problem at the subsystem level is as follows:
  (10) min⁡ f1=−1.508x1x62+7.477x63+0.7854x4x62 s.t.     g3=1.93x43(x2x3x64)−1≤0 g5=A1B1−1100≤0      7.3≤x4≤8.3 2.9≤x6≤3.9      design variables=[x4,x6]      design parameters=[x1,x2,x3].
(3) Power input discipline optimization problem at the subsystem level is as follows:
  (11) min⁡ f2=−1.508x1x72+7.477x73+0.7854x5x72  s.t.     g4=1.93x53(x2x3x74)−1≤0 g6=A2B2−850≤0     7.3≤x5≤8.3 5≤x7≤5.5        design variables=[x5,x7]        design parameters=[x1,x2,x3],

where *A*_1_ = [(745*x*_4_/*x*_2_ *x*_3_)^2^+16.9×10^6^]^0.5^, *B*_1_ = 0.1*x*_6_^3^, *A*_2_ = [(745*x*_5_/*x*_2_ *x*_3_)^2^+157.5×10^6^]^0.5^, and *B*_2_ = 0.1*x*_7_^3^.

This problem is solved at two initial points, (*x*_1_, *x*_2_, *x*_3_, *x*_4_, *x*_5_, *x*_6_, *x*_7_) = (2.65,0.63,18,6.80,6.400,3.00,5.099) and (*x*_1_, *x*_2_, *x*_3_, *x*_4_, *x*_5_, *x*_6_, *x*_7_) = (3.50,0.70,17,7.30,7.715,3.35,5.287). The results are shown in Table 2. In this example, there are only shared design variables in both disciplines and compatibility constraints are not needed in IPO. Thus the optimization problems in IPO are simpler. Compared with CO, IPO enjoys less number of the function calls and higher efficiency than CO in this example.

## 6. Conclusions

In this paper, IPM which is applied in control and coordination in large-scale systems is introduced for MDO problems. Based on the strategy of IPM, IPO is given and its mathematical foundation is presented. Compared with CO method, there are two improvements in IPO. One is that, using the original compatibility constraints in CO, the solutions from the subsystem level are needed to be compared with the target values of discipline design variables and the interaction variables from the system level. If there are a large number of variables, the compatibility constraints will be more complex. It results in more function calls and deteriorates the CO performance. However, using the enhanced compatibility constraints in IPO, only the values of coupled variables from the system level **Y**_*ij*_ and from the discipline analysis ${\hat{Y}}_{ij}^{}$ are needed to be compared during the discipline optimization process. The dimension of variables in the enhanced compatibility constraints is reduced. Thus the enhanced compatibility constraint is simpler than the original compatibility constraint and easer to be applied in the optimization process. The other is that multiple discipline objectives are incorporated and converted to a single objective by an aggregate function in CO. Despite maintaining the discipline autonomy, no discipline objectives are considered at the subsystem level. In IPO, the system objective and the discipline objectives are considered at the system level and the subsystem level, respectively. Thus the framework of IPO is more suitable for the practical engineering.

## Figures and Tables

**Table 1:** Optimization results of the mathematical example.

	Point 1			Point 2		
	MDF	CO	IPO	MDF	CO	IPO
*x*_1_	−0.3001	−0.3257	−0.2999	−0.3000	−0.2190	−0.2993
*x*_2_	0.2999	0.3682	0.2995	0.2998	0.2702	0.2990
*x*_3_	0.8998	0.7870	0.8989	0.8998	0.8706	0.9005
*y*_12_	0.3999	0.3435	0.3996	0.3999	0.4431	0.3995
*y*_21_	0.4999	0.6110	0.4995	0.4998	0.5623	0.4989
*f*	3.6000	3.2212	3.6005	3.6000	3.2560	3.5977
*n*_*s*_	—	74	61	—	77	59
*n*_1_	—	55	41	—	53	45
*n*_2_	—	49	36	—	55	42

**Table 2:** Optimization results of reducer design example.

	Point 1			Point 2		
	MDF	CO	IPO	MDF	CO	IPO
*x*_1_	3.600	3.478	3.600	3.600	3.495	3.472
*x*_2_	0.664	0.630	0.665	0.664	0.644	0.678
*x*_3_	17	18	17	17	17	17
*x*_4_	7.300	7.307	7.300	7.300	7.300	7.300
*x*_5_	7.715	7.781	7.716	7.715	7.715	7.716
*x*_6_	3.351	3.361	3.353	3.351	3.347	3.353
*x*_7_	5.287	5.305	5.287	5.287	5.279	5.287
*f *	2874.360	2851.560	2875.731	2872.068	2744.607	2889.013
*n*_*s*_	—	308	245	—	375	251
*n*_1_	—	354	285	—	319	212
*n*_2_	—	315	255	—	331	273
